# Necrotic Cells Alter IRE1α-XBP1 Signaling and Induce Transcriptional Changes in Glioblastoma

**DOI:** 10.3390/ijms27010474

**Published:** 2026-01-02

**Authors:** Jiwoo Lim, Seulgi Lee, Ye-Seon Hong, Ji Ha Choi, Ala Jo, Jihee Lee Kang, Tae-Jin Song, Youn-Hee Choi

**Affiliations:** 1 Department of Physiology, College of Medicine, Ewha Womans University, Seoul 07804, Republic of Korea; 2 Inflammation-Cancer Microenvironment Research Center, College of Medicine, Ewha Womans University, Seoul 07804, Republic of Korea; 3 Department of Pharmacology, College of Medicine, Ewha Womans University, Seoul 07804, Republic of Korea; 4 Department of Neurology, Seoul Hospital, College of Medicine, Ewha Womans University, Seoul 07804, Republic of Korea; 5 Graduate Programs in System Health Science and Engineering, Ewha Womans University, Seoul 03760, Republic of Korea

**Keywords:** necrosis, glioblastoma, endoplasmic reticulum stress, unfolded protein response, IRE1α-XBP1 signaling

## Abstract

Necrosis is a characteristic feature of glioblastoma multiforme (GBM) and is closely associated with tumor-associated inflammation and poor clinical outcomes. However, the molecular consequences of necrotic cell death on endoplasmic reticulum (ER) stress signaling in GBM cells remain unclear. In this study, we examined the effects of necrotic cells on the ER stress signaling and unfolded protein response (UPR) in human glioblastoma cell lines. Exposure to necrotic cells reduced IRE1α phosphorylation and increased unspliced XBP1 (XBP1u) accumulation, without affecting PERK or ATF6 pathways. These changes were accompanied by enhanced IκBα phosphorylation and impaired autophagic degradation. Treatment with ER stress inducers failed to reverse XBP1u accumulation, and reduced phosphorylation of PKAc was observed together with decreased IRE1α activation. Transcriptomic analysis and quantitative reverse transcription PCR (qRT-PCR) revealed that necrotic cell-induced XBP1u was associated with altered expression of XBP1-related genes, while *XBP1* knockdown produced similar transcriptional changes and enhanced the effects of necrotic cell treatment. These findings suggest that necrotic cells impair canonical IRE1α-XBP1 signaling and induce transcriptional reprogramming in glioblastoma cells, which may contribute to tumor progression.

## 1. Introduction

Astrocytoma is a type of tumor that occurs in the brain or spinal cord and originates from astrocytes, which are the major glial cells of the central nervous system [1]. Glioblastoma multiforme (GBM), classified as grade IV astrocytoma, is the most common and most malignant form of astrocytoma in adults, with a typical median survival of only 12–15 months [2,3]. GBM is highly aggressive, characterized by diffuse infiltration, rapid proliferation, invasion into normal brain parenchyma, and pseudopalisading necrosis, distinguishing it from lower-grade astrocytomas [4]. One hallmark of GBM is tissue necrosis accompanied by microenvironment inflammation [5]. However, the precise mechanisms by which inflammation develops and persists in GBM remain unclear. Despite aggressive treatments, including surgery, chemotherapy, and radiotherapy, GBM prognosis remains poor, with a 5-year survival rate of only 5% and frequent recurrence. Clinical studies have reported a correlation between increased necrosis and worsened patient outcomes [6,7,8].

Necrosis is an accidental form of cell death induced by environmental insults or injuries, such as impaired blood supply, bacterial toxin, or drugs [9]. During necrosis, intracellular contents leak into extracellular space, leading to immune cell infiltration and the establishment of an inflammatory microenvironment. Morphologically, necrosis is characterized by organelle swelling, increased cell volume, plasma membrane disruption, and the release of intracellular contents. These intracellular materials, termed damage-associated molecular patterns (DAMPs), trigger inflammatory response and contribute to disease progression [10]. While necrosis is recognized as a major contributor to inflammation, it may also contribute to cellular stress responses beyond inflammation. In particular, whether necrosis is associated with endoplasmic reticulum (ER) stress in GBM cells remains unclear.

The ER is a multifunctional organelle involved in protein synthesis and folding, lipid biosynthesis, and calcium homeostasis [11]. ER dysfunction results in the accumulation of misfolded proteins, activating a protective signaling network known as the unfolded protein response (UPR). This response aims to restore cellular homeostasis by enhancing chaperone expression, attenuating global protein synthesis, and promoting the degradation of misfolded proteins via three key transmembrane sensors: PERK, IRE1α, and ATF6 [12]. Previous studies have revealed a strong link between ER stress and cancer progression, particularly through UPR activation. Prolonged or unresolved ER stress may result in cell death and tumor suppression [13,14]. Furthermore, tumor-specific microenvironmental stressors, such as hypoxia, nutrient deprivation, pH alterations, and poor vascularization, are known to activate the UPR [15,16,17].

Our recent findings demonstrated that exposure to necrotic conditions induces a secretory phenotype in GBM cells, promoting their migratory and invasive capacities. Based on these findings, the present study aims to investigate whether necrosis influences ER stress and the UPR signaling cascade in GBM cells, with particular attention to whether necrosis leads to subtle alterations in canonical UPR activation.

## 2. Results

### 2.1. The Effect of Necrotic Cells on ER Stress and NF-κB Pathways in Glioblastoma Cells

**Figure 1:**
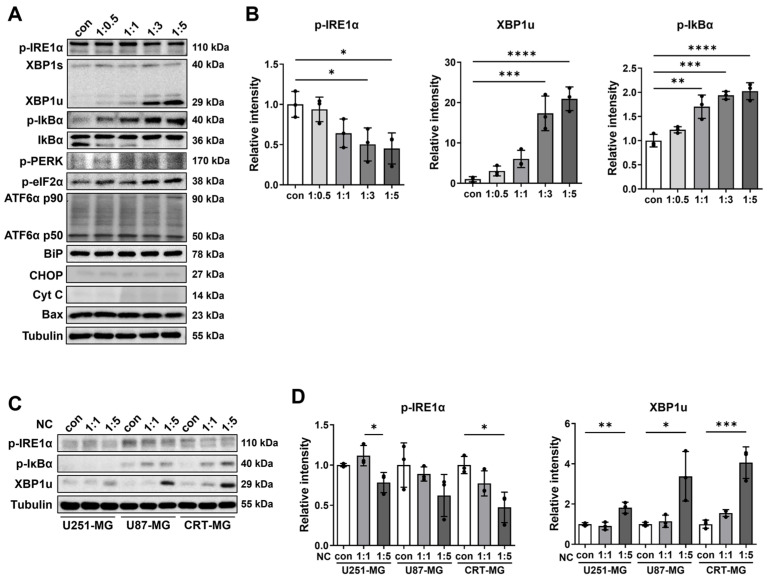
Effects of necrotic cells on ER stress and NF-κB pathways in glioblastoma cells. (**A**) CRT-MG cells were treated with necrotic CRT-MG cells at different ratios (1:0.5–1:5) for 24 h and analyzed by Western blot. (**B**) Quantitative analysis of Western blot band intensities. Data are presented as mean ± SD from three independent experiments. Statistical significance was determined using one-way ANOVA followed by Tukey’s post hoc test. * *p* < 0.05, ** *p* < 0.01, *** *p* < 0.001, **** *p* < 0.0001 vs. control. (**C**) U251-MG, U87-MG, CRT-MG cells were treated with necrotic cells derived from the same cell line (1:5 ratio) and analyzed by Western blot. (**D**) Quantitative analysis of Western blot band intensities. Data are presented as mean ± SD from three independent experiments. Statistical significance was determined using one-way ANOVA followed by Tukey’s post hoc test. * *p* < 0.05, ** *p* < 0.01, *** *p* < 0.001.

To test whether exposure to the necrotic cells induces ER stress in glioblastoma cells, CRT-MG glioblastoma cell line was treated with necrotic CRT-MG, and pathways related to ER stress and NF-κB were assessed by Western blot analysis. The efficiency and reproducibility of necrotic cell preparation were confirmed by flow cytometric analysis (Supplementary Figure S1). Necrotic cell treatment reduced phosphorylation of IRE1α and markedly increased unspliced XBP1 (XBP1u) expression. In parallel, p-IκBα levels increased, while total IκBα decreased, indicating its phosphorylation and degradation in a necrotic cell dose-dependent manner (1:0.5 to 1:5) (Figure 1A). Proteins related to the PERK and ATF6 pathways showed no statistically significant changes upon necrotic cell treatment (Supplementary Figure S2). Exposure to necrotic cells did not induce detectable changes in the expression of mitochondria-related proteins, including cytochrome c and Bax, as assessed by immunoblotting. We next tested whether other glioblastoma cell lines respond similarly. U251-MG, U87-MG, and CRT-MG cells were each treated with necrotic cells derived from their respective cell lines. In U251-MG cells, necrotic cell exposure at the highest ratio (1:5) resulted in a reduction in IRE1α phosphorylation compared with the 1:1 condition, whereas no statistically significant changes were observed in U87-MG cells. In contrast, XBP1u expression was significantly increased at the 1:5 ratio in both U251-MG and U87-MG cells (Figure 1C). Together, these findings suggest that necrotic cell exposure engages the IRE1α–XBP1 axis, leading to the accumulation of XBP1u in glioblastoma cell lines.

### 2.2. Necrotic Cell-Induced XBP1u Accumulation Is Independent of TG- or BFA1-Mediated Pathways

**Figure 2:**
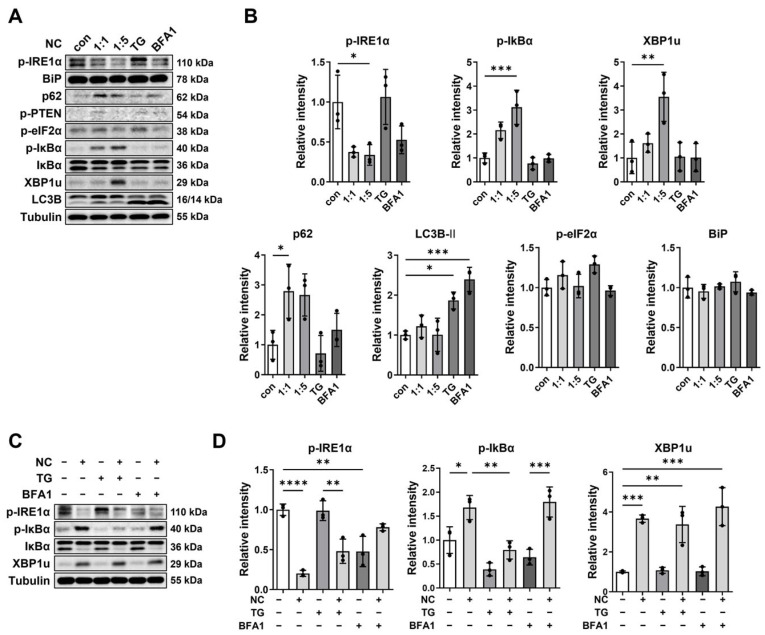
Comparison of necrotic cells with ER stress inducers in CRT-MG cells. (**A**) CRT-MG cells were treated with necrotic cells at different ratios (control, 1:1, and 1:5), thapsigargin (TG, 250 nM), or bafilomycin A1 (BFA1, 10 μM) for 24 h and analyzed by Western blot. LC3B was detected as two bands corresponding to LC3B-I (~16 kDa) and LC3B-II (~14 kDa). (**B**) Quantitative analysis of Western blot band intensities. Data are presented as mean ± SD from three independent experiments. Statistical significance was determined using one-way ANOVA followed by Tukey’s post hoc test. * *p* < 0.05, ** *p* < 0.01, *** *p* < 0.001 vs. control. (**C**) CRT-MG cells were treated with necrotic cells (1:5 ratio) for 24 h and subsequently co-treated with TG (250 nM) or BFA1 (10 μM) for 3 h. Cell lysates were then analyzed by Western blot. (**D**) Quantitative analysis of Western blot band intensities. Data are presented as mean ± SD from three independent experiments. Statistical significance was determined using one-way ANOVA followed by Tukey’s post hoc test. * *p* < 0.05, ** *p* < 0.01, *** *p* < 0.001, **** *p* < 0.0001, as indicated.

We next compared the effects of necrotic cells with those of thapsigargin (TG), a well-known ER stress inducer that can stimulate autophagy, and bafilomycin A1 (BFA1), a well-known autophagic flux inhibitor, focusing on the IRE1α pathway and autophagy-related proteins. Quantitative analysis demonstrated that necrotic cell treatment markedly reduced IRE1α phosphorylation and increased XBP1u expression in CRT-MG cells, consistent with the findings shown in Figure 1 (Figure 2A). In our system, TG treatment did not alter IRE1α phosphorylation, whereas BFA1 decreased it, with XBP1u expression largely unchanged by either treatment. Among the autophagy-related proteins, band intensity analysis revealed that p62 expression—a selective autophagy substrate—was significantly increased in necrotic cell-treated groups, whereas BFA1 treatment did not result in a statistically significant change, suggesting impaired autophagic degradation. In contrast, Western blot signal quantification showed no significant change in LC3B-II following necrotic cell treatment, whereas TG and BFA1 significantly increased LC3B-II levels. Western blot signal quantification showed that phosphorylation of eIF2α and expression of BiP were not significantly altered by necrotic cell treatment, TG, or BFA1. To test the effects of TG and BFA1 on XBP1u levels elevated by necrotic cells, CRT-MG cells were treated with necrotic cells in combination with TG or BFA1. Necrotic cell-induced XBP1u elevation persisted in the presence of TG or BFA1 (lanes 2, 4, and 6, Figure 2C). IRE1α phosphorylation, which was reduced by necrotic cells, showed partial recovery with TG co-treatment but not with BFA1. Phosphorylation of IκBα was increased by necrotic cells, but decreased with TG alone; co-treatment with necrotic cells and TG partially restored IκBα phosphorylation levels compared with TG alone. These results showed that necrotic cell-induced XBP1u accumulation persisted despite TG or BFA1 treatment, indicating that necrotic cells promote IRE1α inactivation and XBP1u accumulation through a mechanism distinct from the pathways targeted by TG or BFA1.

### 2.3. Necrotic Cells Reduce PKAc and IRE1α Phosphorylation, Leading to Persistent XBP1u Accumulation

**Figure 3:**
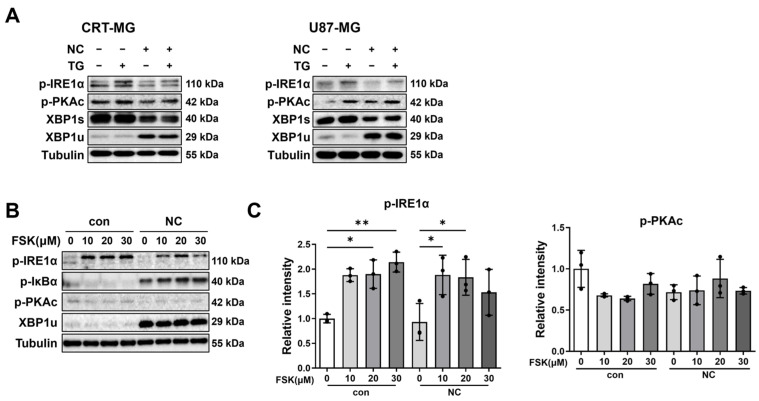
Necrotic cells suppress PKAc and IRE1α activation. (**A**) CRT-MG and U87-MG cells were treated with necrotic cells (1:5 ratio) for 24 h, with or without co-treatment with TG (250 nM) for 3 h, and analyzed by Western blot. (**B**) CRT-MG cells were treated with necrotic cells (1:5 ratio) for 24 h and subsequently co-treated with forskolin (FSK) at the indicated concentrations for 1 h. Cell lysates were analyzed by Western blot. (**C**) Quantitative analysis of Western blot band intensities. Data are presented as mean ± SD from three independent experiments. Statistical significance was determined using one-way ANOVA followed by Tukey’s post hoc test. * *p* < 0.05, ** *p* < 0.01, as indicated.

Because necrotic cells disturb canonical IRE1α downstream signaling and promote XBP1u accumulation, we investigated whether PKAc activity is affected under these conditions. In CRT-MG and U87-MG cells, TG treatment increased the phosphorylation of both PKAc and IRE1α, accompanied by enhanced XBP1 splicing (XBP1s) (Figure 3A, lane 2). In contrast, necrotic cells reduced the phosphorylation of PKAc and IRE1α while increasing XBP1u expression (lane 3). Co-treatment with TG partially restored PKAc and IRE1α phosphorylation, but XBP1u levels remained elevated despite TG treatment (lane 4).

Given the reduction in PKAc phosphorylation by necrotic cells, we next examined whether forskolin (FSK), an adenylyl cyclase activator, could reverse this effect. In CRT-MG cells, FSK alone increased PKAc and IRE1α phosphorylation in a dose-dependent manner (Figure 3B). However, in the presence of necrotic cells, FSK-induced phosphorylation of PKAc and IRE1α was markedly reduced compared with FSK alone. Increased XBP1u expression was detected in necrotic cell-treated groups, and additional treatment with FSK did not affect this induction. These results suggest that necrotic cells diminish PKAc activity, which correlates with reduced IRE1α phosphorylation and contributes to persistent XBP1u accumulation.

### 2.4. XBP1u-Regulated Gene Expression in Necrotic Cell-Treated Glioma Cells

Our data showed that necrotic cells promote XBP1u accumulation by suppressing PKAc and IRE1α phosphorylation. To determine the functional consequence of this accumulation, we next investigated whether XBP1u influences XBP1-dependent gene expression. Since XBP1 normally acts as a transcription factor activated by IRE1α in ER stress, we hypothesized that necrotic cells might alter XBP1 target gene expression independently of canonical ER stress activation.

**Figure 4:**
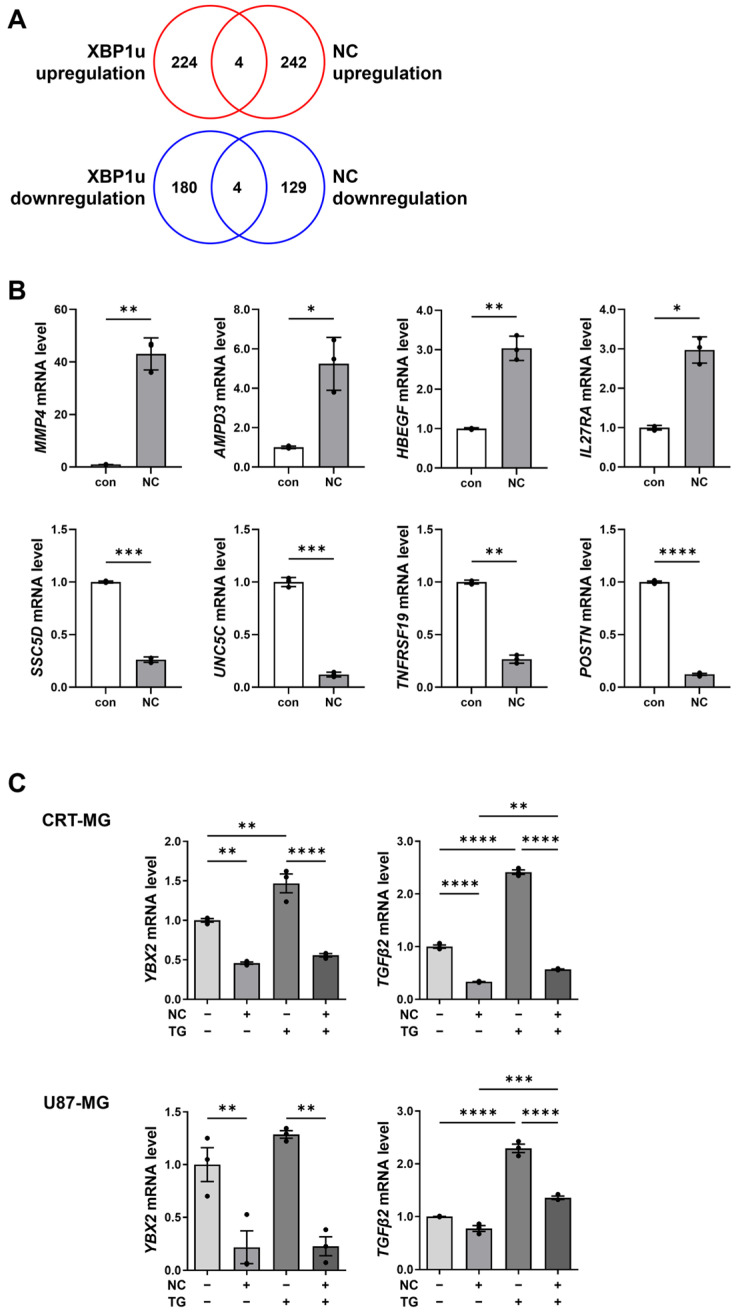
XBP1u-regulated gene expression in necrotic cell-treated glioblastoma cells. (**A**) Venn diagram showing the overlap of differentially expressed genes between necrotic cell-treated glioblastoma cells and HeLa cells overexpressing XBP1u. (**B**) quantitative reverse transcription PCR (qRT-PCR) validation of the upregulated (MMP4, AMPD3, HBEGF, IL27RA) and downregulated (UNC5C, SSC5D, TNFRSF19, POSTN) genes in CRT-MG cells treated with necrotic cells (1:5 ratio) for 24 h. Data are presented as mean ± SD from three independent experiments. Statistical significance was determined using Student’s *t*-test. * *p* < 0.05, ** *p* < 0.01, *** *p* < 0.001, **** *p* < 0.0001 vs. control (**C**) CRT-MG and U87-MG cells were treated with necrotic cells (1:5 ratio) for 24 h, with or without co-treatment with TG (250 nM) for 3 h, and mRNA expression of XBP1-related genes was measured by qRT-PCR. Data are presented as mean ± SD from three independent experiments. Statistical significance was determined using one-way ANOVA followed by Tukey’s post hoc test. ** *p* < 0.01, *** *p* < 0.001, **** *p* < 0.0001, as indicated.

We compared a published dataset of gene expression changes in HeLa cells overexpressing XBP1u with our previous transcriptome data from necrotic cell-treated glioma cells [18]. Venn diagram analysis revealed 4 commonly upregulated and 4 commonly downregulated genes between the two datasets (Figure 4A). qRT-PCR validation in CRT-MG cells confirmed that *MMP4*, *AMPD3*, *HBEGF*, and *IL27RA* were upregulated, while *UNC5C*, *SSC5D*, *TNFRSF19*, and *POSTN* were downregulated by necrotic cell treatment (Figure 4B). These results indicated that, even without XBP1u overexpression, necrotic cell-induced XBP1u accumulation is sufficient to regulate the expression of XBP1u-associated genes.

We further examined the expression of XBP1-related genes *YBX2* and *TGFβ2* in the presence of TG. TG treatment increased *YBX2* and *TGFβ2*, whereas necrotic cells reduced their expression in both CRT-MG and U87-MG cells (Figure 4C). Co-treatment with TG and necrotic cells modestly increased *YBX2* and *TGFβ2* expression compared with necrotic cell treatment alone. Collectively, these findings suggest that necrotic cells modulate XBP1-related gene expression through XBP1u accumulation, likely contributing to altered transcriptional programs in glioma cells.

### 2.5. XBP1 Knockdown and Necrotic Cell Treatment Exert Additive Effects on XBP1-Related Gene Expression

**Figure 5:**
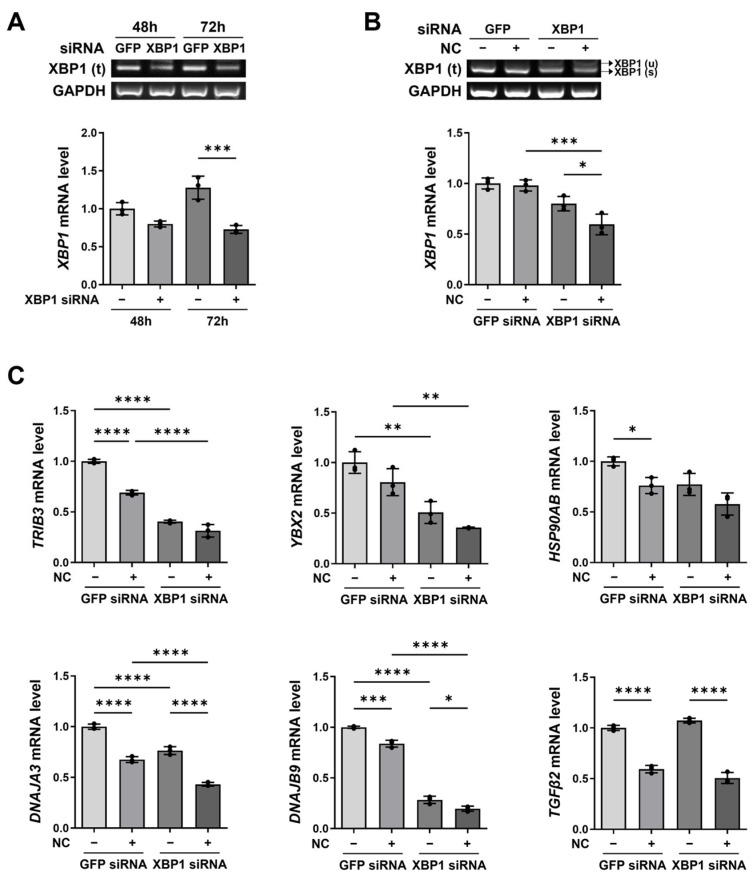
Effects of XBP1 knockdown and necrotic cell treatment on XBP1 expression and XBP1-related genes. (**A**) U87-MG cells were transfected with XBP1 siRNA or control GFP siRNA for 48 or 72 h. Knockdown efficiency was assessed by RT-PCR, and relative XBP1 mRNA levels were measured by qRT-PCR. Data are presented as mean ± SD from three independent experiments. Statistical significance was determined using one-way ANOVA followed by Tukey’s post hoc test. *** *p* < 0.001, as indicated. (**B**) U87-MG cells were transfected with XBP1 siRNA for 72 h and treated with necrotic cells (1:5 ratio) for 24 h. XBP1 expression was assessed by RT-PCR and qRT-PCR. Data are presented as mean ± SD from three independent experiments. Statistical significance was determined using one-way ANOVA followed by Tukey’s post hoc test. * *p* < 0.05, *** *p* < 0.001, as indicated. (**C**) U87-MG cells were transfected with XBP1 siRNA or control GFP siRNA for 72 h and treated with necrotic cells (1:5 ratio) for 24 h. Expression of XBP1-related genes was analyzed by qRT-PCR. Data are presented as mean ± SD from three independent experiments. Statistical significance was determined using one-way ANOVA followed by Tukey’s post hoc test. * *p* < 0.05, ** *p* < 0.01, *** *p* < 0.001, **** *p* < 0.0001, as indicated.

To determine whether *XBP1* knockdown produces similar effects to necrotic cell treatment, we silenced *XBP1* using siRNA and confirmed knockdown efficiency by RT-PCR. *XBP1* mRNA levels were reduced following siRNA transfection, with greater knockdown observed at 72 h compared to 48 h (Figure 5A). In necrotic cell-treated cultures, *XBP1* siRNA further decreased *XBP1* mRNA expression compared with knockdown alone (Figure 5B).

We next examined whether necrotic cells and *XBP1* knockdown affect the expression of XBP1-related genes (Figure 5C). In U87-MG cells transfected with control *GFP* siRNA, necrotic cell treatment reduced the expression of XBP1-related genes. In the absence of necrotic cells, *XBP1* knockdown significantly decreased the expression of most XBP1-related genes, except *TGFβ2*. Furthermore, combined treatment with necrotic cells and *XBP1* siRNA led to a more pronounced reduction in XBP1-related gene expression than either treatment alone. These results indicate that necrotic cells and *XBP1* knockdown exert similar suppressive effects on XBP1-related gene expression.

## 3. Discussion

Glioblastoma multiforme (GBM) is the most aggressive form of glioma and the most common primary brain tumor in adults. One of the distinguishing features of GBM compared with other gliomas is the frequent presence of necrosis. Previous studies have mainly focused on the molecular mechanisms underlying necrosis formation; however, the consequences of necrosis on surrounding GBM cells remain largely unclear. Our previous studies demonstrated that necrotic cells induce the secretion of several chemokines, including interleukin (IL)-8, MCP1, and MIP-3α, which promote GBM cell migration, invasion, and microglial infiltration [3,19]. In the present study, we investigated whether necrosis is associated with endoplasmic reticulum (ER) stress and unfolded protein response (UPR) signaling in GBM and explored the role of necrosis in modulating XBP1 signaling and downstream gene expression.

Our results demonstrated that necrotic cells suppress IRE1α phosphorylation in a dose-dependent manner, leading to XBP1u accumulation and IκBα phosphorylation. This indicates that necrosis impairs the canonical IRE1α-XBP1s pathway while favoring XBP1u accumulation. Consistent with previous reports that cancer cells often exhibit defective ER stress signaling [20], we found that thapsigargin (TG) treatment enhanced IRE1α and PKAc phosphorylation and upregulated XBP1 target genes such as *YBX2* and *TGFβ2*. In contrast, our results showed that necrotic cells diminished a part of ER stress signaling and reduced the expression of XBP1-related genes. Similar effects were observed with *XBP1* knockdown, supporting the possibility that XBP1u contributes to necrosis-driven transcriptional changes by antagonizing the transcription-factor function of XBP1s, thereby yielding outcomes comparable to *XBP1* knockdown. We further confirmed that both *XBP1* knockdown and necrotic cells treatment reduced the expression of multiple XBP1-related genes, with greater suppression observed when combined. These findings suggest that XBP1u accumulation may contribute to transcriptional changes driven by necrotic cells which lead to UPR dysfunction in GBM cells by uncoupling IRE1α activation from proper downstream transcriptional regulation. Such alterations may shift the balance from pro-apoptotic to pro-survival pathways, which could contribute to GBM malignancy, rapid progression, and poor prognosis.

The present study has several limitations. First, although glioblastoma is characterized by extensive necrosis, precise necrotic-to-viable cell ratios at the cellular level are difficult to define in vivo. Accordingly, the necrotic cell ratios used in this study were intended to model increasing necrotic burden in a controlled in vitro setting and to evaluate dose-dependent effects on IRE1α phosphorylation and XBP1u accumulation, rather than to directly recapitulate in situ cellular proportions. Second, the upstream mechanisms by which necrotic cells influence viable tumor cells remain unresolved. In this study, we did not experimentally determine whether these effects arise from uptake of necrotic cell-derived materials or from receptor-mediated signaling triggered by soluble damage-associated molecular patterns released from necrotic cells. Given that necrotic cells release a heterogeneous mixture of intracellular components, isolating and functionally validating individual mediators remains technically challenging [21,22]. Third, necrotic cells were generated exclusively from glioblastoma cell lines. In the tumor microenvironment, dying cells can also include immune and stromal populations, which may elicit distinct responses in neighboring tumor cells; therefore, our conclusions should be interpreted in the context of tumor cell line–derived necrotic cells.

IRE1α has been implicated in broader translational control through the eIF4F complex in other cancer contexts [23,24]; however, the present study did not examine global protein synthesis or metabolic regulation. Previous studies have demonstrated that chronic ER stress and IRE1α–XBP1 signaling contribute to metabolic adaptation, immune modulation, and autophagy-mediated stress tolerance in glioblastoma [25,26,27,28]. In this context, our findings raise the possibility that necrosis-associated stress may represent an upstream factor capable of influencing ER stress–dependent programs within tumor cells. Further investigation using models that incorporate metabolic and immune components of the tumor microenvironment will be required to address these questions.

The UPR is typically an adaptive response that restores ER homeostasis through increased chaperone expression, inhibition of protein synthesis, and removal of misfolded proteins [29]. In normal cells, activation of PERK, ATF6, and IRE1α pathways coordinates these functions [30]. However, our results show that in GBM cells exposed to necrosis, PERK and ATF6 signaling were not significantly affected, whereas IRE1α activity was strongly suppressed. This imbalance led to XBP1u accumulation and suppression of XBP1s-dependent gene expression, reflecting a dysfunctional UPR. Similar dysregulation of ER chaperones and UPR components has been reported in various cancers, including breast, liver, lung, ovarian, and pancreatic cancers, as well as glioblastoma [31,32,33,34,35,36]. Our study extends these findings by demonstrating that the alteration of IRE1α–XBP1 signaling we identified may contribute to the dysregulation of ER chaperones and UPR components observed in cancers in part, and that necrosis may serve as a critical factor exacerbating UPR dysfunction in GBM.

Taken together, our results suggest that exposure of cancer cells to necrotic cells disrupts IRE1α–PKAc signaling and promotes XBP1u accumulation, thereby impairing UPR function in GBM. ER stress inducers such as thapsigargin did not reverse necrosis-induced XBP1u accumulation, indicating that this process is controlled by a mechanism distinct from canonical UPR signaling and may involve a previously unrecognized pathway. Although our findings imply that necrotic signals suppress IRE1α and induce XBP1 dysfunction, the upstream regulator responsible for these effects remains unidentified and warrants further investigation. The resulting IRE1α–XBP1 dysfunction provides a potential explanation for the dysregulation of ER chaperones and UPR components commonly observed in cancers. Accordingly, targeting the necrosis-XBP1u accumulation and underappreciated mechanism of UPR disruption in GBM may provide a foundation for future therapeutic exploration to restore ER function and attenuate GBM aggressiveness.

## 4. Materials and Methods

### 4.1. Cell Culture and Preparation of Necrotic Cells

Human glioblastoma cell lines CRT-MG, U251-MG, and U87-MG were cultured in Dulbecco’s Modified Eagle’s Medium (DMEM; WelGENE, Inc., Daegu, Republic of Korea) supplemented with 10% fetal bovine serum (FBS; WelGENE), 2 mM L-glutamine, 100 U/mL penicillin, and 10 μg/mL streptomycin. Cells were maintained at 37 °C in a humidified, 5% CO_2_. Necrotic cells were generated by subjecting cultures to five cycles of freezing in liquid nitrogen and thawing in a 37 °C water bath. Quantification of necrotic cells was performed by flow cytometry using Annexin V–FITC and propidium iodide (PI) staining, as previously described [19], to determine the proportions of PI-positive necrotic cells and Annexin V–positive apoptotic cells. This analysis was repeated for each preparation to confirm the reproducibility of necrotic cell generation.

### 4.2. Reagents and Antibodies

Thapsigargin (TG; T9033), bafilomycin A1 (BFA1; B1793), and forskolin (FSK; F6886) were obtained from Sigma-Aldrich (St. Louis, MO, USA). Antibodies against p-PKAc (#4781), p-IκB-α (#2859), BiP (#3183), LC3B (#2775), CHOP (#2895), p62 (#88588), p-eIF2α (#9721), p-PERK (#3179), and p-PTEN (#9551) were purchased from Cell Signaling Technology (Danvers, MA, USA). Antibodies against IκBα (#sc-371), ATF-6α (#sc-166659), and Bax (#sc-493) were purchased from Santa Cruz Biotechnology (Dallas, TX, USA). Antibodies against cytochrome C (#ab13575), X-box binding protein-1 (XBP1; #ab37152), and unspliced XBP1 (XBP1u; #ab109221) were purchased from Abcam (Cambridge, UK). Antibodies against α-tubulin (#T5168) and p-IRE1α (#PA1-16927) were purchased from Sigma-Aldrich and Thermo Fisher Scientific (Waltham, MA, USA), respectively. Horseradish peroxidase (HRP)-conjugated secondary antibodies were obtained from Thermo Fisher Scientific.

### 4.3. Western Blot Analysis

Western blotting was performed as previously described with minor modifications [37]. Cells were incubated with or without necrotic cells for 24 h and lysed in RIPA buffer containing protease inhibitor cocktail (GenDEPOT, Katy, TX, USA). Lysates were centrifuged at 13,000 rpm for 30 min at 4 °C, and supernatants were subjected to immunoblotting. Equal amounts of protein were separated by SDS-PAGE and transferred to polyvinylidene difluoride (PVDF) membranes. Membranes were blocked with 5% skim milk in TBS-T buffer (0.1% Tween-20) for 1 h at room temperature, incubated overnight at 4 °C with primary antibodies (1:1000). After incubation with primary antibodies, membranes were incubated with species-specific HRP-conjugated secondary antibodies (anti-rabbit IgG-HRP and anti-mouse IgG-HRP, 1:3000). Immunoreactive bands were visualized using an LAS-4000mini imaging system (GE Healthcare, Chicago, IL, USA). Protein bands were visualized with enhanced chemiluminescence (ECL; Amersham, Buckinghamshire, UK). Band intensity was quantified using ImageJ software (version 1.54, NIH, Bethesda, MD, USA).

### 4.4. Reverse Transcription Polymerase Chain Reaction (RT-PCR)

Total RNA was extracted using Easy-BLUE^TM^ reagent (iNtRON Biotechnology, Gyeonggi-do, Republic of Korea) according to tshe manufacturer’s instructions. cDNA was synthesized from 1 μg of total RNA using ReverTra Ace qPCR RT master Mix (Toyobo, Osaka, Japan). RT-PCR was carried out using a thermal cycler (Bio-Rad Laboratories, Hercules, CA, USA). PCR products were separated on 2.5% agarose gels stained with GelRed^TM^ (Biotium, Fremont, CA, USA) and visualized using Gel Doc^TM^ XT+ System (Bio-Rad Laboratories, Hercules, CA, USA). Primer sequences are listed in Table 1.

### 4.5. Quantitative Real-Time Polymerase Chain Reaction (qRT-PCR)

For qRT-PCR, cDNA was synthesized as described above. Reactions were performed on an ABI StepOnePlus Real-time PCR machine (Applied Biosystems, Foster City, CA, USA) using Power SYBR^TM^ Green PCR Master Mix (Applied Biosystems) according to the manufacturer’s protocol. Relative gene expression was calculated using the comparative Ct (∆∆Ct) method with GAPDH as an internal control. The primer sequences were listed in Table 1.

### 4.6. siRNA Transfection

Cells were seeded at a density of 2 × 10^5^ cells/well in 6-well plates on the day before transfection. Cells were transfected with either human XBP1 siRNA (Bioneer, Daejeon, Republic of Korea) or control green fluorescence protein (GFP) siRNA using Lipofectamine RNAiMAX reagent (Invitrogen, Carlsbad, CA, USA) according to the manufacturer’s instructions, at a final concentration of 200 nM. Cells were incubated in complete medium for 48 h or 72 h prior to treatment with necrotic cells. Knockdown efficiency was confirmed by RT-PCR and qRT-PCR.

### 4.7. Statistical Analysis

All experiments were independently repeated at least three times. Data are presented as mean ± standard deviation (SD). Statistical analyses were performed using GraphPad Prism 10 (GraphPad Software, San Diego, CA, USA). Comparisons between two groups were conducted using Student’s *t*-test, while comparisons among more than two groups were analyzed using one-way analysis of variance (ANOVA) followed by Tukey’s post hoc test. Differences were considered statistically significant at *p* < 0.05.

## Figures and Tables

**Table 1:** List of primer sequence use for RT-PCR and qRT-PCR analysis.

	Gene (Human)		Sequence	Tm (°C)
RT-PCR primer	XBP1	Forward	5′-CCTGGTTGCTGAAGAGGAGG-3′	60.04
		Reverse	5′-CCATGGGGAGATGTTCTGGAG-3′	59.86
	GAPDH	Forward	5′-TGGAAATCCCATCACCATCT-3′	56.17
		Reverse	5′-GTCTTCTGGGTGGCAGTGAT-3′	59.67
qRT-PCR primer	DNAJA3	Forward	5′-AGGAGGGATTCCTTTCCAAACTTA-3′	59.39
		Reverse	5′-TCTGGAATCCTCCCGTCTCC-3′	60.40
	DNAJB9	Forward	5′-GCCATGAAGTACCACCCTGACA-3′	62.27
		Reverse	5′-TCGTCTATTAGCATCTGAGAGTGT-3′	58.87
	HSP90AB	Forward	5′-TTGGGTATCGGAAAGCAAGCC-3′	60.95
		Reverse	5′-GTGCACTTCCTCAGGCATCTTG-3′	61.77
	TGFβ2	Forward	5′-ACACTCAGCACAGCAGGGTCCT-3′	65.86
		Reverse	5′-TTGGGACACGCAGCAAGGAGAAG-3′	65.32
	TRIB3	Forward	5′-AGCGGTTGGAGTTGGATGACA-3′	61.99
		Reverse	5′-GGCCACAGCAGTTGCACGA-3′	63.75
	YBX2	Forward	5′-GATGTCGTGGAAGGAGAGAAGG-3′	60.16
		Reverse	5′-GATGAATCGGCGGGACTTACGT-3′	62.72
	MPP4	Forward	5′-TAGCCAGCAGATGGTGTACGTC-3′	62.10
		Reverse	5′-GGTCCACAATCTGGAGGATGTC-3′	60.42
	AMPD3	Forward	5′-CTGGTTCATCCAGCACAAGGTC-3′	61.45
		Reverse	5′-GGCAGGAAGATGTTCTCCAGGA-3′	61.75
	HBEGF	Forward	5′-TGTATCCACGACCAGCTGCTA-3′	60.96
		Reverse	5′-TGCTCCTCCTTGTTTGGTGTGG-3′	62.98
	UNC5C	Forward	5′-AGAATGGAGGCAAGGACTGCGA-3′	63.98
		Reverse	5′-GCCAGGCAAACGATCACTGCTA-3′	63.44
	SSC5D	Forward	5′-TGTGACTGCCAGTGTTCTGGAG-3′	62.44
		Reverse	5′-GGAGTGTTTCGTGGTTGGCATC-3′	62.27
	TNFRSF19	Forward	5′-GGTGCATTCTGCAGCCAGTCTT-3′	63.67
		Reverse	5′-CAGGCATCTGAAAACTCGCCAC-3′	62.32
	POSTN	Forward	5′-CAGCAAACCACCTTCACGGATC-3′	62.02
		Reverse	5′-TTAAGGAGGCGCTGAACCATGC-3′	63.46
	IL27RA	Forward	5′-GTGTGGGTATCAGGGAACCTCT-3′	61.16
		Reverse	5′-TCCTTCTGGACTCAGCTCACGA-3′	62.79
	GAPDH	Forward	5′-GAGTCAACGGATTTGGTCGT-3′	58.21
		Reverse	5′-AATGAAGGGGTCATTGATGG-3′	55.35

## Data Availability

The original contributions presented in this study are included in the article/Supplementary Materials. Further inquiries can be directed to the corresponding authors.

## Supplementary Materials

The following supporting information can be downloaded at: https://www.mdpi.com/article/10.3390/ijms27010474/s1.

## References

[1] Kiray H., Lindsay S.L., Hosseinzadeh S., Barnett S.C. (2016). The multifaceted role of astrocytes in regulating myelination. Exp. Neurol..

[2] Furnari F.B., Fenton T., Bachoo R.M., Mukasa A., Stommel J.M., Stegh A., Hahn W.C., Ligon K.L., Louis D.N., Brennan C. (2007). Malignant astrocytic glioma: Genetics, biology, and paths to treatment. Genes Dev..

[3] Jung Y., Ahn S.H., Park H., Park S.H., Choi K., Choi C., Kang J.L., Choi Y.H. (2018). MCP-1 and MIP-3alpha Secreted from Necrotic Cell-Treated Glioblastoma Cells Promote Migration/Infiltration of Microglia. Cell. Physiol. Biochem..

[4] Rong Y., Durden D.L., Van Meir E.G., Brat D.J. (2006). ‘Pseudopalisading’ necrosis in glioblastoma: A familiar morphologic feature that links vascular pathology, hypoxia, and angiogenesis. J. Neuropathol. Exp. Neurol..

[5] Waters M.R., Gupta A.S., Mockenhaupt K., Brown L.N., Biswas D.D., Kordula T. (2019). RelB acts as a molecular switch driving chronic inflammation in glioblastoma multiforme. Oncogenesis.

[6] Barboriak D.P., Provenzale J.M. (2002). Evaluation of software for registration of contrast-enhanced brain MR images in patients with glioblastoma multiforme. AJR Am. J. Roentgenol..

[7] Holland E.C. (2000). Glioblastoma multiforme: The terminator. Proc. Natl. Acad. Sci. USA.

[8] Raza S.M., Lang F.F., Aggarwal B.B., Fuller G.N., Wildrick D.M., Sawaya R. (2002). Necrosis and glioblastoma: A friend or a foe? A review and a hypothesis. Neurosurgery.

[9] Fulda S., Gorman A.M., Hori O., Samali A. (2010). Cellular stress responses: Cell survival and cell death. Int. J. Cell Biol..

[10] Yang Y., Jiang G., Zhang P., Fan J. (2015). Programmed cell death and its role in inflammation. Mil. Med. Res..

[11] Chaudhari N., Talwar P., Parimisetty A., Lefebvre d’Hellencourt C., Ravanan P. (2014). A molecular web: Endoplasmic reticulum stress, inflammation, and oxidative stress. Front. Cell. Neurosci..

[12] Yadav R.K., Chae S.W., Kim H.R., Chae H.J. (2014). Endoplasmic reticulum stress and cancer. J. Cancer Prev..

[13] Corazzari M., Gagliardi M., Fimia G.M., Piacentini M. (2017). Endoplasmic Reticulum Stress, Unfolded Protein Response, and Cancer Cell Fate. Front. Oncol..

[14] Garg A.D., Agostinis P. (2014). ER stress, autophagy and immunogenic cell death in photodynamic therapy-induced anti-cancer immune responses. Photochem. Photobiol. Sci..

[15] Wang H., Pezeshki A.M., Yu X., Guo C., Subjeck J.R., Wang X.Y. (2014). The Endoplasmic Reticulum Chaperone GRP170: From Immunobiology to Cancer Therapeutics. Front. Oncol..

[16] Koumenis C. (2006). ER stress, hypoxia tolerance and tumor progression. Curr. Mol. Med..

[17] Fels D.R., Koumenis C. (2006). The PERK/eIF2alpha/ATF4 module of the UPR in hypoxia resistance and tumor growth. Cancer Biol. Ther..

[18] Gebert M., Sobolewska A., Bartoszewska S., Cabaj A., Crossman D.K., Kroliczewski J., Madanecki P., Dabrowski M., Collawn J.F., Bartoszewski R. (2021). Genome-wide mRNA profiling identifies X-box-binding protein 1 (XBP1) as an IRE1 and PUMA repressor. Cell. Mol. Life Sci..

[19] Ahn S.H., Park H., Ahn Y.H., Kim S., Cho M.S., Kang J.L., Choi Y.H. (2016). Necrotic cells influence migration and invasion of glioblastoma via NF-kappaB/AP-1-mediated IL-8 regulation. Sci. Rep..

[20] Zhang W., Shi Y., Oyang L., Cui S., Li S., Li J., Liu L., Li Y., Peng M., Tan S. (2024). Endoplasmic reticulum stress-a key guardian in cancer. Cell Death Discov..

[21] Murao A., Aziz M., Wang H., Brenner M., Wang P. (2021). Release mechanisms of major DAMPs. Apoptosis.

[22] Schuermans S., Kestens C., Marques P.E. (2024). Systemic mechanisms of necrotic cell debris clearance. Cell Death Dis..

[23] Ron D. (2002). Translational control in the endoplasmic reticulum stress response. J. Clin. Investig..

[24] Robert F., Roman W., Bramoulle A., Fellmann C., Roulston A., Shustik C., Porco J.A., Shore G.C., Sebag M., Pelletier J. (2014). Translation initiation factor eIF4F modifies the dexamethasone response in multiple myeloma. Proc. Natl. Acad. Sci. USA.

[25] Kesarwani P., Kant S., Prabhu A., Chinnaiyan P. (2017). The interplay between metabolic remodeling and immune regulation in glioblastoma. Neuro-Oncology.

[26] Simpson J.E., Gammoh N. (2020). The impact of autophagy during the development and survival of glioblastoma. Open Biol..

[27] Gu D., Hu L., Yang K., Yuan W., Shan D., Gao J., Li J., Gimple R.C., Dixit D., Zhu Z. (2025). Stress-induced pro-inflammatory glioblastoma stem cells secrete TNFAIP6 to enhance tumor growth and induce suppressive macrophages. Dev. Cell.

[28] Medikonda R., Abikenari M., Schonfeld E., Lim M. (2025). The Metabolic Orchestration of Immune Evasion in Glioblastoma: From Molecular Perspectives to Therapeutic Vulnerabilities. Cancers.

[29] Almanza A., Carlesso A., Chintha C., Creedican S., Doultsinos D., Leuzzi B., Luis A., McCarthy N., Montibeller L., More S. (2019). Endoplasmic reticulum stress signalling—From basic mechanisms to clinical applications. FEBS J..

[30] Chen X., Shi C., He M., Xiong S., Xia X. (2023). Endoplasmic reticulum stress: Molecular mechanism and therapeutic targets. Signal Transduct. Target. Ther..

[31] Alberti G., Vergilio G., Paladino L., Barone R., Cappello F., Conway de Macario E., Macario A.J.L., Bucchieri F., Rappa F. (2022). The Chaperone System in Breast Cancer: Roles and Therapeutic Prospects of the Molecular Chaperones Hsp27, Hsp60, Hsp70, and Hsp90. Int. J. Mol. Sci..

[32] Samanta S., Tamura S., Dubeau L., Mhawech-Fauceglia P., Miyagi Y., Kato H., Lieberman R., Buckanovich R.J., Lin Y.G., Neamati N. (2020). Clinicopathological significance of endoplasmic reticulum stress proteins in ovarian carcinoma. Sci. Rep..

[33] Robinson C.M., Talty A., Logue S.E., Mnich K., Gorman A.M., Samali A. (2021). An Emerging Role for the Unfolded Protein Response in Pancreatic Cancer. Cancers.

[34] Rangel D.F., Dubeau L., Park R., Chan P., Ha D.P., Pulido M.A., Mullen D.J., Vorobyova I., Zhou B., Borok Z. (2021). Endoplasmic reticulum chaperone GRP78/BiP is critical for mutant Kras-driven lung tumorigenesis. Oncogene.

[35] Ajoolabady A., Kaplowitz N., Lebeaupin C., Kroemer G., Kaufman R.J., Malhi H., Ren J. (2023). Endoplasmic reticulum stress in liver diseases. Hepatology.

[36] Simbilyabo L.Z., Yang L., Wen J., Liu Z. (2024). The unfolded protein response machinery in glioblastoma genesis, chemoresistance and as a druggable target. CNS Neurosci. Ther..

[37] Lim J., Choi J.H., Park E.M., Choi Y.H. (2020). Interaction of promyelocytic leukemia/p53 affects signal transducer and activator of transcription-3 activity in response to oncostatin M. Korean J. Physiol. Pharmacol..

