# Supplementary material for: Necrotic Cells Alter IRE1α-XBP1 Signaling and Induce Transcriptional Changes in Glioblastoma
Source: Int J Mol Sci. 2026 Jan 2;27(1):474. doi: 10.3390/ijms27010474 (PMC12786997; doi:10.3390/ijms27010474)
Supplement: Supplementary file 1 [file ijms-27-00474-s001.zip › ijms-4039964-supplementary.pdf]

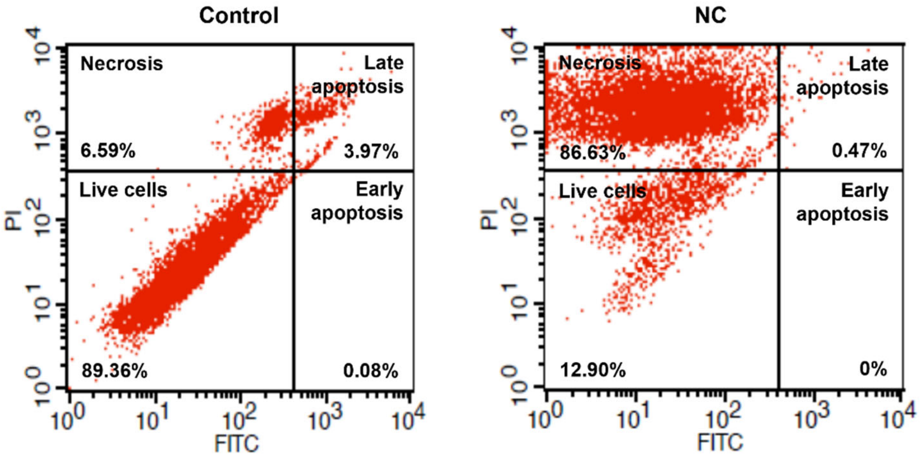

Supplementary Figure S1. Flow cytometric characterization of necrotic cell preparation.

CRT-MG cells were subjected to five freeze–thaw cycles using liquid nitrogen and stained with Annexin V–FITC and propidium iodide (PI). Flow cytometric analysis demonstrated that more than 85% of cells were consistently PI-positive, indicating necrosis, while Annexin V–positive apoptotic cells were minimally detected. Representative data from independent preparations are shown.

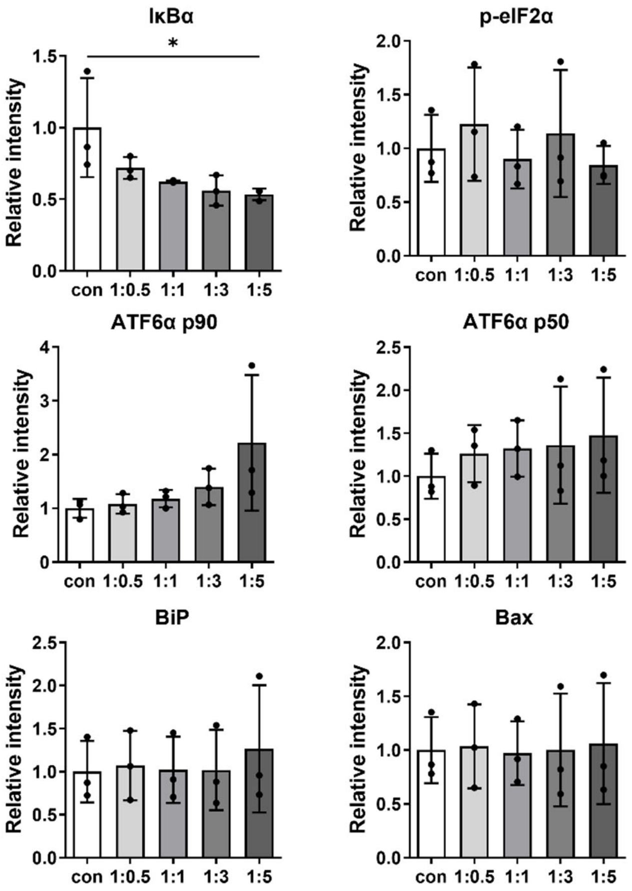

Supplementary Figure S2. Quantification of additional Western blot data shown in Figure 1A.

Band intensities of PERK- and ATF6 pathway–related proteins, mitochondria-associated proteins, and IκBα were quantified and normalized to the loading control. Data are presented as mean ± SD with individual data points (n = 3 independent experiments). Statistical analysis was performed as described in the Methods.
